# RETRACTION: Role of Inhibitors of Apoptosis Proteins in Testicular Function and Male Fertility: Effects of Polydeoxyribonucleotide Administration in Experimental Varicocele

**DOI:** 10.1155/bmri/9825079

**Published:** 2026-04-28

**Authors:** BioMed Research International

RETRACTION: L. Minutoli, S. Arena, P. Antonuccio, C. Romeo, A. Bitto, C. Magno, M. Rinaldi, A. Micali, N. Irrera, G. Pizzino, F. Galfo, F. Squadrito, D. Altavilla, H. Marini, “Role of Inhibitors of Apoptosis Proteins in Testicular Function and Male Fertility: Effects of Polydeoxyribonucleotide Administration in Experimental Varicocele,” *BioMed Research International*, 2015, 248976, https://doi.org/10.1155/2015/248976

The above article, published online on 04 August 2015 in Wiley Online Library (wileyonlinelibrary.com), has been retracted by agreement between the journal Editor‐in‐Chief, Yoel Klug; and John Wiley & Sons Ltd. The retraction has been agreed due to concerns identified with the figures [1, 2]. Specifically:

• In Figure 4, there are unexpected, repeated regions between panels A and E.
• In Figure 2b, the 2nd and 6th NAIP bands appear similar when rotated 180 degrees

Additionally, ethics concerns have been raised regarding the treatment of the animals used in the study [2], and the authors could not provide the ethical approval documentation.

After assessment of the concerns and the author’s response, the editorial board have confirmed the retraction of the article due to loss of confidence in the reliability of the results and the ethics concerns.

The authors did not respond to the retraction.
